# Enhancing global control of alcohol to reduce unsafe sex and HIV in sub-Saharan Africa

**DOI:** 10.1186/1744-8603-5-16

**Published:** 2009-11-17

**Authors:** Matthew F Chersich, Helen V Rees, Fiona Scorgie, Greg Martin

**Affiliations:** 1 International Centre for Reproductive Health, Department of Obstetrics and Gynaecology, Ghent University, Ghent, Belgium; 2 Department of Obstetrics and Gynaecology, Ghent University, Ghent, Belgium; 3 London School of Hygiene and Tropical Medicine, London, UK; 4 Population Council, Johannesburg, South Africa

## Abstract

Sub-Saharan Africa carries a massive dual burden of HIV and alcohol disease, and these pandemics are inextricably linked. Physiological and behavioural research indicates that alcohol independently affects decision-making concerning sex, and skills for negotiating condoms and their correct use. More than 20 studies in Africa have reported higher occurrence of HIV among people with problem drinking; a finding strongly consistent across studies and similar among women and men. Conflation of HIV and alcohol disease in these setting is not surprising given patterns of heavy-episodic drinking and that drinking contexts are often coterminous with opportunities for sexual encounters. HIV and alcohol also share common ground with sexual violence. Both perpetrators and victims of sexual violence have a high likelihood of having drunk alcohol prior to the incident, as with most forms of violence and injury in sub-Saharan Africa. Reducing alcohol harms necessitates multi-level interventions and should be considered a key component of structural interventions to alleviate the burden of HIV and sexual violence. Brief interventions for people with problem drinking (an important component of primary health care), must incorporate specific discussion of links between alcohol and unsafe sex, and consequences thereof. Interventions to reduce alcohol harm among HIV-infected persons are also an important element in positive-prevention initiatives. Most importantly, implementation of known effective interventions could alleviate a large portion of the alcohol-attributable burden of disease, including its effects on unsafe sex, unintended pregnancy and HIV transmission.

## Introduction

Globally, HIV and other sexually-transmitted infections (STI) account for 6.3% of the burden of disease and alcohol for 4%, similar to that caused by tobacco (4.1%) and high blood pressure (4.4%) [1]. In some sub-Saharan countries, such as South Africa, for example, the burden attributable to these conditions is even greater - HIV and other STI constitute about a third of disease and alcohol an estimated 7.9% [2]. Overall, much of sub-Saharan Africa carries a massive burden of HIV and of alcohol disease, and these pandemics are inextricably linked. The conditions share many common determinants and together exacerbate the underlying socio-economic inequalities in this region. As we discuss in this article, alcohol disease and HIV have an especially intimate link: alcohol has independent effects on decision-making concerning sex, and on skills for negotiating condoms and their correct use. Thus far global initiatives to prevent HIV and other sexually transmitted infections (STI) have largely ignored the potential mediatory role of alcohol in unsafe sex (for example, note that the list of WHO HIV prevention priorities does not mention alcohol) [3].

Alcohol use results in a considerable range of diseases, the occurrence of which is contingent upon three factors: lifetime cumulative volume consumed; patterns of drinking; and drinking contexts [4,5]. Overall lifetime volume of alcohol is linked to chronic social problems (such as unemployment) and to chronic diseases such as alcoholic liver cirrhosis. By contrast, pattern of drinking (amount per drinking episode), in particular frequent episodes of intoxication, is a powerful mediator of acute problems such as accidents, interpersonal violence and high-risk sexual behaviour [5,6]. Context of alcohol use is also a critical determinant of its consequences, as opportunities for sexual encounters and for drinking alcohol often co-exist in both social dynamics and physical locations [7-10]. This means that the impact of alcohol use on acute social behaviours (including sexual behaviours [11]) is shaped more by the *'how' *and *'where' *of alcohol consumption, [10] than by the frequency of drinking or cumulative lifetime volume of alcohol.

Unlike settings with low-risk drinking patterns (classically southern European patterns of drinking with meals), sub-Saharan Africa is characterised by harmful patterns of drinking. This includes the use of large quantities of alcohol per occasion, but also drinking to intoxication in public spaces, heavy drinking during cultural festivals and drinking outside of mealtimes [12,13]. These patterns often take the form of weekend binge drinking [6,13] and are true of both rural and urban areas, and across all social strata [14,15]. It follows that acute rather than chronic alcohol problems predominate in sub-Saharan Africa, and include road traffic accidents, crime, interpersonal violence and unsafe sex, afflicting harm to self and others. Globally, drinking alcohol has been linked with an increased number of sexual partners, regretted sexual relations, inconsistent condom use, condom accidents and an increased incidence of STI [6,16-19]. Studies in sub-Saharan Africa, in particular [8], have found strong associations between alcohol consumption and unprotected sex, early sexual debut, multiple sex partners and having an STI [6,8,11,20].

HIV and alcohol also share common ground with sexual violence. A person's risk of rape or of perpetrating rape increases during heavy drinking episodes. Both perpetrators and victims of sexual violence have a high likelihood of having drunk alcohol prior to the incident, possibly because alcohol intoxication makes the drinker an easier target for potential perpetrators [21].

More than 20 studies in Africa have reported higher occurrence of HIV among people with problem drinking [8,11,20,22,23]. A meta-analysis of these studies found that compared with non-drinkers, non-problem drinkers had 1.6 fold higher HIV prevalence, while problem drinkers had a 2.0 fold higher prevalence [23]. This finding is strongly consistent across studies and is similar among women and men.

Challenging evidence for a causal relationship between alcohol, unsafe sex and HIV, are some studies which suggest that personality factors such as impulsivity or sensation seeking, as well as contextual factors confound the alcohol and sex relationship [24-26]. It must be acknowledged that the relationship between alcohol use and risky sex is complex and that associations between HIV and alcohol use may, at least in part, be accounted for by the fact that heavy drinkers are inherently different from other population groups [27,28]. Overall, however, in recent years the causal pathway between alcohol intoxication, unsafe sex and HIV acquisition has become more clearly defined and the evidence increasingly compelling [9].

**Figure 1:**
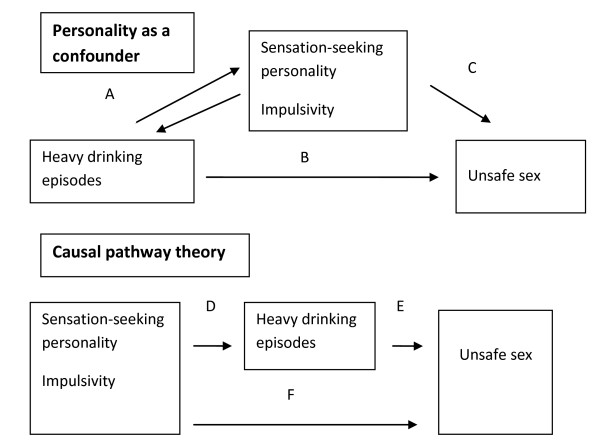
**Do personality types confound associations between alcohol and unsafe sex or is alcohol an intermediate factor on the causal pathway between personality and unsafe sex?**. In the confounding theory, the association between heavy drinking episodes and unsafe sex (arrow B) is explained by the confounding factor of personality types (arrows A). In this model, a reduction in heavy drinking would have no effect on unsafe sex as personality types are independently associated with unsafe sex (Arrow C).   In the causal pathway theory, heavy drinking is an intermediary factor in the causal pathway between personality types and unsafe sex (arrow D plus E). Prevention of heavy episodic drinking would reduce the portion of unsafe sex attributable to arrow E, though will have no effect on the contribution of arrow F.

An argument can be made that alcohol is an intermediate factor on the causal pathway between certain personality types and unsafe sex (see figure 1). Thus people with impulsive personalities, for example, may still have unsafe sex when not influenced by alcohol, just as this group of people may drive dangerously when sober. However, reducing harmful drinking in this population would alleviate a portion of their unsafe sex, just as curbs on drinking and driving reduce a substantial portion of their road traffic accidents. Although few would accept the theory that personality types completely explain the association between alcohol use and outcomes such as road traffic accidents, crime and sexual violence, more evidence is required to determine whether associations between alcohol and unsafe sex are confounded by personality types or if alcohol mediates this relationship.

## Alcohol control measures, within the scope of HIV prevention

Reducing alcohol harms necessitates both population and individual-level interventions, which will have marked health and social benefits beyond a reduction in the burden of HIV, sexual violence and unintended pregnancy. Policies such as increased taxation and limitations on the availability of alcohol, which are highly effective in lowering alcohol harm, are often strongly contested by the alcohol industry [29]. Not unlike the tobacco industry, the alcohol industry shifts the onus of alcohol control towards individuals who are encouraged to adopt "responsible behaviour", and away from controls on alcohol marketing and availability [30]. Interestingly, the alcohol industry, through its marketing efforts and easy availability, does its utmost to subvert the ability of individuals to be responsible. By analogy, it's like a parent encouraging an obese child to take responsibility for their diet, but then stocking the fridge with chocolates and pasting posters depicting the glamour of sugar on the wall.

Given the substantial contribution of the industry to the GDP of many countries [31], the alcohol industry can almost certainly yield substantial political power. Yet, while it is recognized that control of alcohol is difficult and politically charged, increased interventions to control harmful use of alcohol are urgently needed.

Universal community-wide interventions can increase knowledge of the harmful effects of alcohol use and change community norms and values [32,33]. It is critical that these interventions make explicit links between alcohol use, unsafe sex and HIV. Policies and interventions targeting establishments which serve alcohol are especially important because of the overlap between drinking and the dense sexual networks in these contexts [11]. Implementing HIV prevention activities within drinking venues, such as bars, beer halls and taverns could reach large numbers of persons at greatest risk for HIV infection. Importantly, evidence indicates that these interventions are feasible [34]. Condoms can be made accessible within drinking venues, for example, with minimal disruption to retail activities and can be promoted with simple messaging using media such as posters or stickers. Though general public education and warning labels on drinks are obvious interventions and generally uncontested by the alcohol industry, they are poorly effective in their current form [35]. Evidence from use of warning labels in tobacco control suggests that the use of more graphic and larger warnings can influence behaviour.

The health sector has a critical role to play in mitigating alcohol-related harm, especially in the form of selective alcohol interventions, such as Brief Interventions for people with problem drinking. The term "Brief Intervention" refers to time-limited, patient-centred counselling that focuses on changing patient behaviour and has been shown to empower people to control their alcohol use and its effects on sexual behaviour [36]. Such services are an important component of primary health care, and can be configured to include specific discussion of links between alcohol and unsafe sex and consequences thereof. Research in Kenya has shown that alcohol-HIV counselling interventions can be included within services provided at HIV voluntary counselling and testing centres [37]. In part, this intervention is feasible because routine provider-initiated screening for alcohol use is analogous to provider-initiated HIV testing. Here, the AUDIT screening interview or similar tools could be used to identify whether a person has hazardous drinking (patterns of drinking that increase the risk of harmful consequences for the user and others), harmful drinking (alcohol consumption that results in consequences to social, physical and mental health) or alcohol dependence (behavioural, cognitive, and physiological phenomena, such as increased alcohol tolerance and a physical withdrawal reaction when alcohol is discontinued) [38]. While alcohol treatment services are indicated for people who are alcohol dependant, individuals with hazardous or harmful drinking patterns require Brief Interventions or similar services, which are highly cost effective [39]. These Brief Interventions fill the key gap between community-level prevention efforts and more intensive treatment for persons who are physically or psychologically dependant on alcohol. Access to public-sector services for substance abuse treatment is low in resource-constrained settings, with inadequate government funding of these services [40]. Overall, with alcohol use, as with sexual behaviour, promotion of *safer *behaviours and contexts is more effective than promotion of total abstinence.

Adolescents, sex workers, migrant labourers and those who work in the transport industry are particularly vulnerable to the effects of alcohol on sexual behaviour and require targeted interventions. Interventions to reduce alcohol harm among HIV-infected persons are also an important element in positive-prevention initiatives. A study in Cape Town, South Africa, showed that almost one in five adults with HIV infection also have alcohol abuse or dependence [41]. Similar findings have been reported in other countries [42,43]. HIV-positive men, among whom heavy drinking before sex remains common, are in particular need of intervention [41,44]. Adoption of safer drinking patterns would have marked benefits for themselves, but also would assist them to enact safer sex decisions and reduce risk of further transmission of HIV to their partners [45,46].

## Priority interventions for mitigating the impact of alcohol on unsafe sex in sub-Saharan Africa

• **Measure the proportion of unsafe sex that is attributed to alcohol, and consequently what portion of the burden of HIV and other STI, sexual violence and unintended pregnancy is attributable to alcohol use**. Even though global figures already show that alcohol harm is high, they underestimate its effects as they exclude the considerable social harms of alcohol [5]. Though it is difficult to make precise estimates at a population-level of the effects of alcohol on social behaviours and on an individual's interaction with their sexual partner(s) and family, this does not justify ignoring such effects; especially in high HIV burden countries. Inclusion of standardised measures of alcohol use within future national household surveys could provide data necessary for estimating alcohol-attributable fractions.

• **Establish global mechanisms for alcohol control, similar to those used for tobacco control**. Lessons could be learnt from the control of other potentially harmful substances, such as infant formula and tobacco.

• **HIV-alcohol links must be clearly articulated within national strategies for HIV prevention and for alcohol control**. Given the burden of HIV in many African countries and causal links with alcohol, drunk sex should be considered as dangerous as drunk driving or tobacco use and warrants equal preventive efforts. HIV-alcohol links must therefore be clearly articulated within national HIV prevention strategies, but also within alcohol control policies. The South African government HIV and STI National Strategic Plan 2007-2011 places alcohol control among the key interventions required to reduce sexual transmission of HIV [47].

• **Develop clear public messaging that unsafe alcohol use can lead to HIV transmission**. In addition to alcohol-HIV messaging on warning labels on drinks for example, combining alcohol and sex topics within educational initiatives among youth appears effective [48-50]. Soul City, a South African multi-media health promotion project, has frequently focused on the relationship between alcohol and HIV, especially in its popular television series [51].

• **Restrict advertising of alcohol**. This is analogous to bans on tobacco advertising, and could include banning alcohol adverts on billboards, or allowing TV alcohol adverts only after 21:00 to avoid youth being exposed to alcohol marketing [33].

• **Raise taxes on alcohol**. This reduces alcohol harm, increases government revenue and assists in recouping the costs of alcohol harm. Levels of alcohol taxation in South Africa are still below international standards, and lower than the neighbouring country Botswana which is pioneering alcohol control in the region [52]. Increases in taxation of alcohol in South Africa are only marginally above inflation rates [53] and there remains little evidence that a reduction in national alcohol consumption is a prominent policy goal for the South African government [54]. Taxation is especially effective among young drinkers who are most price sensitive.

• **Health-care providers should screen for hazardous or harmful use of alcohol at the time of diagnosing HIV and in later visits for HIV care and treatment**. As an HIV diagnosis commonly leads to changes in sexual behaviour, this is a critical opportunity to provide Brief Interventions or similar services to further support adoption of safer sexual practices. Both HIV testing and receipt of ART are key life stages or transition points for many people, and signify important moments of opportunity for intervention by health workers [46].

• **Large-scale research is needed to document whether HIV incidence is lowered by interventions to reduce high-risk drinking, as well as the feasibility of changing drinking patterns and making drinking contexts safer in sub-Saharan Africa**. Though evidence suggests that adoption of safer drinking patterns may reduce unsafe sex [55-58], additional research is needed to demonstrate that HIV incidence is lowered by alcohol-reduction interventions. By satisfying the important reversibility criteria of causality, this evidence is important for more definitively substantiating a causal association between alcohol and HIV. A large trial assessing this is warranted, given the major policy implications of such a finding.

## Conclusion

A substantial proportion of unsafe sex occurs due to alcohol-related disinhibition, diminished rational capacity and impaired decision-making. Reduction in cognitive restraint with alcohol use also hampers skills for condom negotiation and their correct use. For communities and individuals in sub-Saharan Africa, interventions are thus needed to change the way alcohol is used, so that hazardous drinking patterns are shifted to safer patterns.

Control of alcohol use, including alcohol-sex linkages, has been prioritised by the World Health Assembly [59]. International frameworks for alcohol control are now required, building on lessons learnt in tobacco control. Urgency for prevention interventions is clear, as are the drinking venues where such interventions are most needed. Campaigns against alcohol harm may take time to bring about change, but are equally essential as more immediate priorities for HIV prevention, such as condom promotion. Far-reaching structural measures like alcohol control create the conditions necessary for achieving sustained HIV prevention results. Implementing these measures to reduce the chronic burden of alcohol and its mediatory effects on unsafe sex and HIV will take political courage, but could have notable long-range effects.
